# Supplementary figures and images for: A Novel, LAT/Lck Double Deficient T Cell Subline J.CaM1.7 for Combined Analysis of Early TCR Signaling
Source: Cells. 2021 Feb 6;10(2):343. doi: 10.3390/cells10020343 (PMC7915312; doi:10.3390/cells10020343)

## LAT

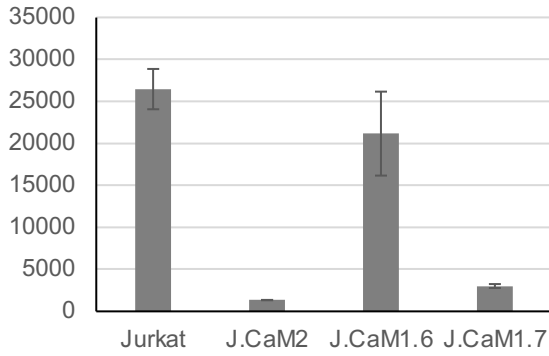

## Lck

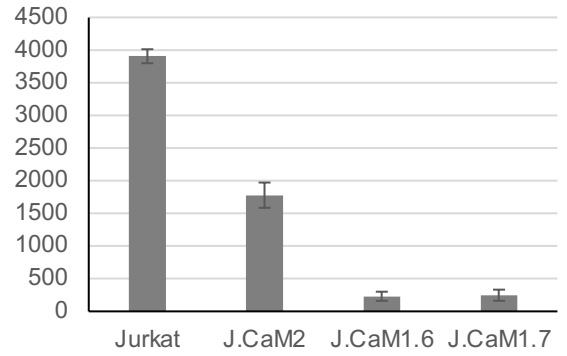

## ZAP-70

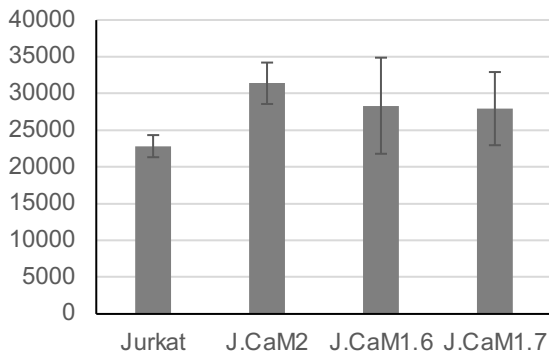

## Grb2

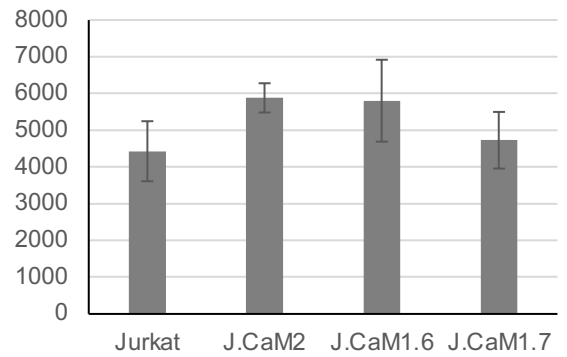

## $\beta$ -actin

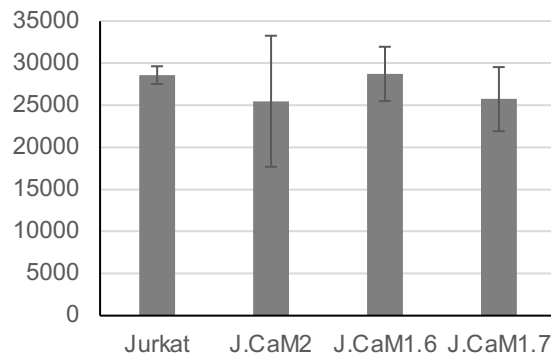

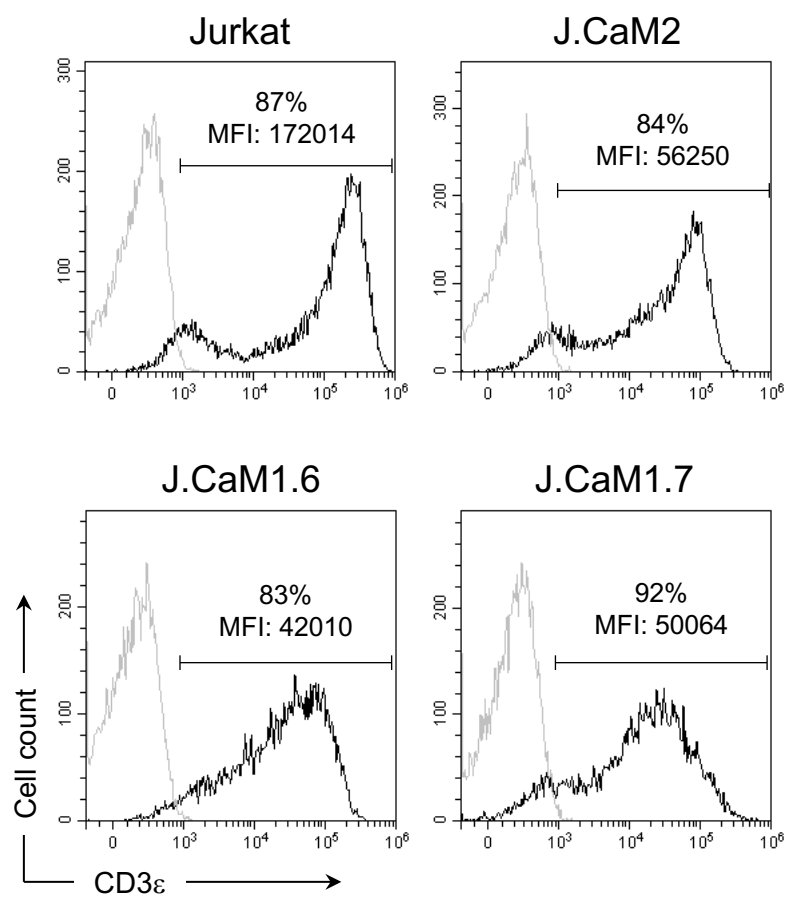

Supplementary Figure S2

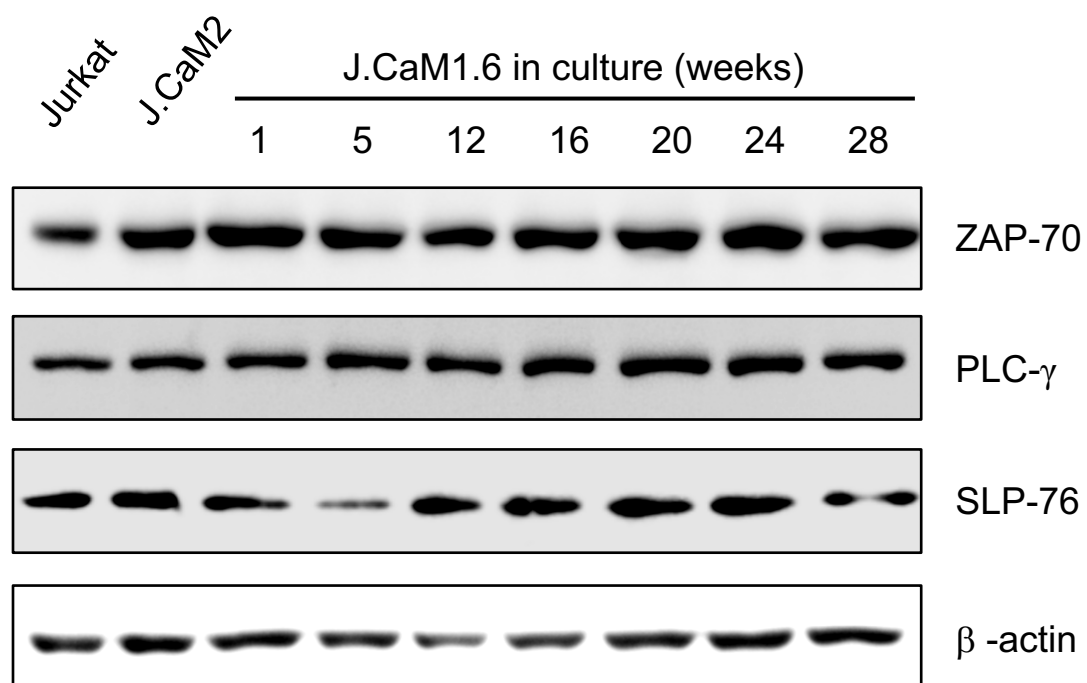

Supplementary Figure S3

LAT-P-Y171

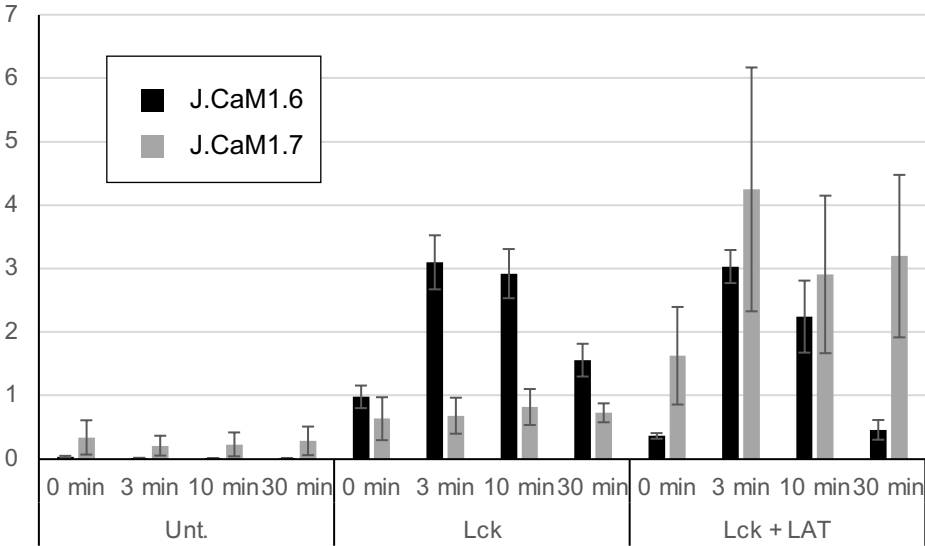

P-PLC- $\gamma$

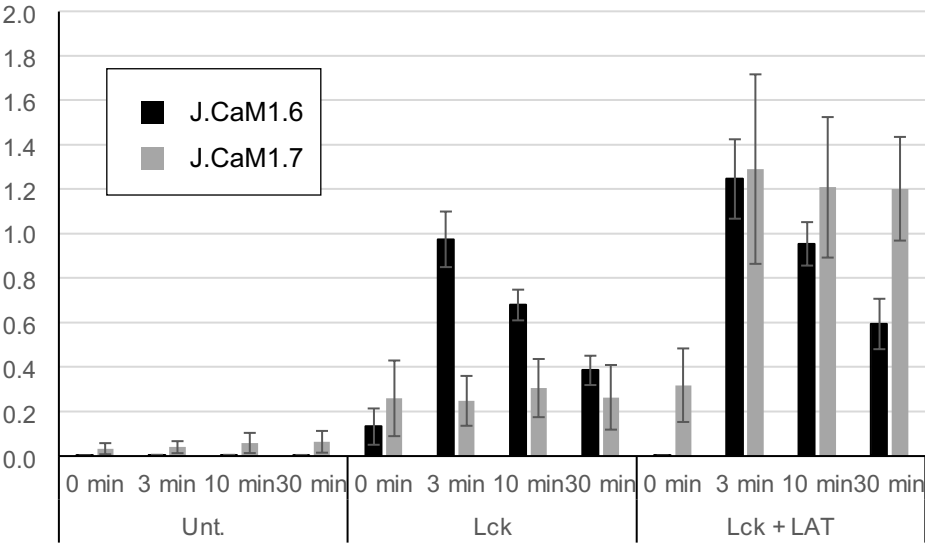

P-Erk

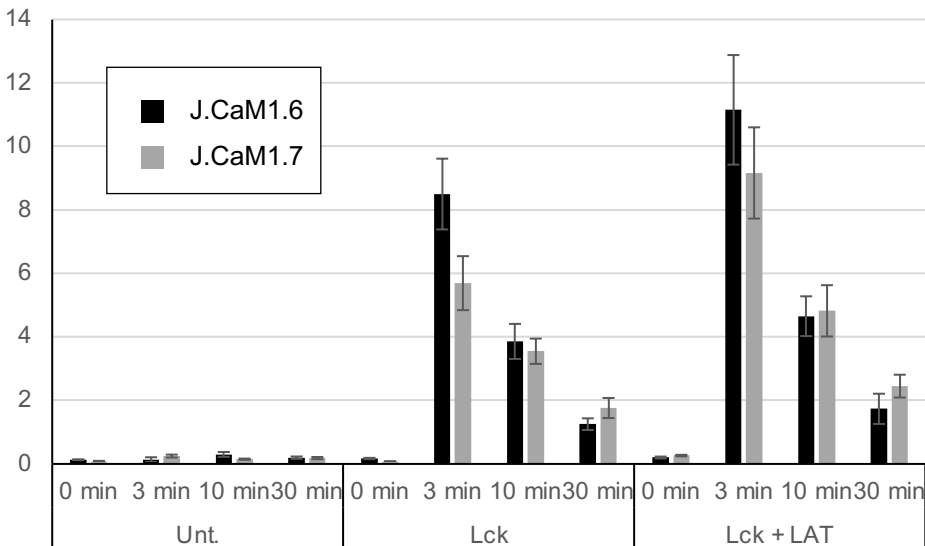

Supplement: Supplementary file 1 [file cells-10-00343-s001.pdf]
